# The oxygen delivery response to acute hypoxia during incremental knee extension exercise differs in active and trained males

**DOI:** 10.1186/1476-5918-7-11

**Published:** 2008-08-12

**Authors:** Michael D Kennedy, Darren ER Warburton, Carol A Boliek, Ben TA Esch, Jessica M Scott, Mark J Haykowsky

**Affiliations:** 1 Faculty of Rehabilitation Medicine, University of Alberta, Edmonton, Alberta, Canada; 2 Cardiovascular Physiology and Rehabilitation Laboratory, University of British Columbia, Vancouver, British Columbia, Canada

## Abstract

**Background:**

It is well known that hypoxic exercise in healthy individuals increases limb blood flow, leg oxygen extraction and limb vascular conductance during knee extension exercise. However, the effect of hypoxia on cardiac output, and total vascular conductance is less clear. Furthermore, the oxygen delivery response to hypoxic exercise in well trained individuals is not well known. Therefore our aim was to determine the cardiac output (Doppler echocardiography), vascular conductance, limb blood flow (Doppler echocardiography) and muscle oxygenation response during hypoxic knee extension in normally active and endurance-trained males.

**Methods:**

Ten normally active and nine endurance-trained males (VO_2max _= 46.1 and 65.5 mL/kg/min, respectively) performed 2 leg knee extension at 25, 50, 75 and 100% of their maximum intensity in both normoxic and hypoxic conditions (FIO_2 _= 15%; randomized order). Results were analyzed with a 2-way mixed model ANOVA (group × intensity).

**Results:**

The main finding was that in normally active individuals hypoxic sub-maximal exercise (25 – 75% of maximum intensity) brought about a 3 fold increase in limb blood flow but decreased stroke volume compared to normoxia. In the trained group there were no significant changes in stroke volume, cardiac output and limb blood flow at sub-maximal intensities (compared to normoxia). During maximal intensity hypoxic exercise limb blood flow increased approximately 300 mL/min compared to maximal intensity normoxic exercise.

**Conclusion:**

Cardiorespiratory fitness likely influences the oxygen delivery response to hypoxic exercise both at a systemic and limb level. The increase in limb blood flow during maximal exercise in hypoxia (both active and trained individuals) suggests a hypoxic stimulus that is not present in normoxic conditions.

## Background

A number of investigators have reported that during hypoxic sub-maximal knee extension (KE) exercise an increase in limb blood flow, leg oxygen extraction and enhanced limb vascular conductance occurs compared to the equivalent work rate in normoxia [1-4]. The effect of hypoxia on cardiac output (Q), total vascular conductance and muscle oxygenation during incremental KE exercise is not as well understood [2,4-7]. Collectively, these results indicate that compensation for the reduced arterial oxygen saturation in response to hypoxia occurs to a greater extent at the level of the exercising limb. However the aforementioned results have not compared trained versus active participant's response to acute hypoxic exercise.

Highly trained athletes have a greater stroke volume, total vascular conductance and leg oxygen extraction during normoxic exercise compared to normally active individuals [8,9]. However, limited research has examined the comparative cardiovascular response of highly trained athletes to active individuals during acute hypoxic maximal exercise [10,11]. This is surprising considering the number of mass athletic competitions held at altitude (trail running races, cross country skiing races, mountain bike races) that include both average and elite participants. In addition, the increasing practice of altitude exposure in the form of tents for home use and live high – train low training methods purported to improve athletic performance [12-15], necessitates the need for more research examining the cardiovascular changes to acute hypoxia in well trained and lesser trained participants.

Since little research has made a comparison of training status for both central and peripheral components of O_2 _delivery it was reasonable to assume that a differential response to acute hypoxia would occur based on training status of the participants. However, how this differential response might be manifested remains unclear. Thus our aim was to determine the changes in the central (lungs and heart) and peripheral (limb blood flow and muscle oxygenation) cardiovascular response to acute hypoxia in incremental knee extension exercise between active and well trained males. To assess the full O_2 _cascade muscle oxygenation was measured to provide an assessment of O_2 _delivery and utilization [16,17] within muscle microcirculation whereby muscle microcirculation is considered to be the terminal end of O_2 _delivery [18]. Further, there is good evidence that large muscle mass exercise such as cycling at maximum exercise intensity is blunted in acute hypoxia compared to normoxic conditions in both active and trained males and females [10,19,20]. Thus, in an effort to make a direct comparison at both sub-maximal and maximal exercise in hypoxia compared to normoxia, KE exercise (which is not limited by O_2 _supply) was chosen as the mode of exercise [3,21]. It was hypothesized that hypoxia would affect a greater cardiovascular and skeletal muscle oxygenation response compared to normoxia and that the magnitude of the response to hypoxia would be similar in trained and active males.

## Methods

### Participants Characteristics

Nine highly trained and 10 normally active males served as participants in this study (see descriptive data provided in Table 1). The highly trained participants were required to have a VO_2max _> 65 mL/kg/min and a minimum of 5 years of endurance training. The active participants had a VO_2max _< 50 mL/kg/min but no specific endurance training requirement. The study received full ethical approval for human subjects by both the Clinical Research Ethics Board (University of British Columbia) and the Health Research Ethics Board (University of Alberta). All participants were recruited from the Vancouver area and written informed consent was obtained from all participants.

**Table 1:** Participant characteristics.

	Trained (N = 9)	Active (N = 10)
Age (yrs)	25.3 ± 4.0	28.0 ± 3.4
Height (cm)	185.7 ± 3.8	180.7 ± 6.8
Weight (kg)	79.8 ± 5.7	79.8 ± 8.6
Body fat (%)	8.2 ± 2.2	11.0 ± 4.7
Hematocrit	45.3 ± 3.8	43.5 ± 3.0
Absolute VO_2max _(L/min)	5.3 ± 0.2	3.7 ± 0.6
Relative VO_2max _(mL/kg/min)	65.5 ± 3.5	46.1 ± 4.5
Peak power output (Watts)	486.7 ± 34.0	354.0 ± 48.6

Values are means ± SD for each group.

### Study Design

Maximal aerobic power (VO_2max_) was determined on an electronically-braked cycle ergometer. In addition participants underwent a graded bilateral KE exercise test to fatigue using a custom built weight machine. During these tests expired gas analysis was acquired using a commercially available metabolic cart (Physio-Dyne, Max-1, AEI Technologies, Naperville IL). Participants then performed, on a subsequent day, bilateral KE sub-maximal and maximal exercise (25, 50, 75 and 100% of peak power) under normoxic and hypoxic (15% O_2_) conditions. Participants were blinded to the condition (hypoxia or normoxia) and half of the participants completed the hypoxic condition first and half completed the normoxic condition first. The hypoxic condition was induced by breathing bottled gas fed into a 200 L gas reservoir that was humidified by bubbling the gas through a 40 L water bottle. Participants started breathing hypoxic gas for approximately 5 minutes after which baseline measures were recorded while subjects sat quietly in the seated position for another 2 – 5 minutes. The hypoxic gas mixture was breathed continuously for the duration of the hypoxic session.

#### Graded Exercise Tests

The VO_2max _test was performed by participants starting at 0 watts and increasing 30 watts per minute at 80 – 85 revolutions per minute (rpm) until volitional exhaustion. The primary criteria for attainment of VO_2max _was volitional exhaustion as well as 1 of the following additional criteria: (1) a plateau or decrease in VO_2 _with increasing intensity; (2) attainment of age predicted maximum heart rate (220 – age); and (3) respiratory exchange ratio > 1.15. All participants stopped their VO_2max _test due to volitional fatigue and all participants met at least one of the other additional criteria for attainment of VO_2max_. The KE exercise was performed on a standard weight training machine used to perform KE exercise. This KE machine differs from custom or modified KE devices used in previous investigations in two ways: 1) it required more muscular effort and 2), the movement primarily involved the quadriceps with little hamstrings involvement. The graded bilateral KE test was performed by fastening the participant's ankles to the bar of the KE machine with a starting position of the knee at approximately 90° from horizontal, where the subject moved the weight through a range of approximately 80°. Subjects were allowed to grasp stabilization bars on either side of the seat to reduce any contribution of non-knee extensor muscle activity to the exercise. The bar to which the subject's ankles were attached was adjustable to accommodate the different lower leg lengths of the participants. After resting baseline measures were confirmed participants exercised for the first minute at a cadence of 40 contractions per minute moving just the KE bar, which weighed approximately 2.3 kg (equal to 4 – 6.5 watts of intensity depending on the length of the KE bar). Every subsequent minute 1.14 kg (3 watts) of weight was added while maintaining a cadence of 40 contractions per minute. The test was stopped when the participant could no longer consistently maintain a cadence of 40 contractions per minute. The cadence was chosen based on pilot testing where 40 contractions per minute allowed for full of range of motion especially at the heavy workloads.

#### Relative Load Tests

The contraction frequency for the KE loads was 40 contractions per minute. The duration of each workload was 3–5 minutes for the sub-maximal workloads (25 – 50 – 75%) and approximately 3 minutes at the maximal workload. The exact duration was partly determined by the time it took for stabilization of oxygen consumption and heart rate and the amount of time needed to record all measures. There were no participants that were unable to sustain similar length of workload in hypoxia compared to the normoxic condition. Rest breaks of 2 minutes between loads were provided for all workloads. The loads were ordered from easiest to hardest and included loads of 25, 50, 75, and 100% of maximum intensity based on the graded exercise test to fatigue. This equated to average watts at 25, 50, 75 and 100% relative intensity of 24, 43, 59 and 78 watts for the active group and 32, 59, 85, and 111 watts for the trained group.

### Measurements

#### Ventilation, Oxygen Consumption, Heart Rate, Oxyhemoglobin Saturation and Hematocrit

Ventilation and oxygen consumption were continuously monitored using a computerized metabolic measurement cart (Physio-Dyne, Max-1, AEI Technologies, Naperville IL) and recorded breath by breath and then averaged every minute using Physio-Dyne Metabolic System software. The Physio-Dyne Max 1 metabolic measurement system has been shown to be a reliable and valid method for expired gas analysis [22] and was specifically chosen because it allows manual calibration with a reported accuracy of the oxygen analyser from 0 – 100%. Gas analyzers were calibrated with gases of known concentration which spanned the range of inspired O_2 _concentrations used for this study before each experiment and the pneumotach (Hans-Rudolph no. 8300, Kansas City, MO) was calibrated with a 3-L syringe. Heart rate was transmitted and recorded to the metabolic cart wirelessly (Polar Electro Oy, Kempele, Finland). Oxyhemoglobin saturation was measured by a pulse oximeter (Ohmeda Biox 3740, Louisville, CO) and hematocrit was determined on the first test day, from a finger prick puncture with standardized techniques and measurement [23].

#### Stroke Volume, Cardiac Output, Total Vascular Conductance, and Blood Pressure

To estimate ascending aorta blood flow velocity a 1.9-Mhz continuous wave Doppler transducer was positioned in the suprasternal notch. The velocity time integral (VTI) was recorded by tracing the velocity curve for individual beats off-line. VTI values for the 4 curves with greatest consistent values and most distinct spectral envelopes were averaged at rest as well within the final minute of each load. Aortic area was calculated at rest from measurements of the maximal diameter (mid-systole) at the level of the aortic valve hinge points from 2-D echocardiography (parasternal long-axis view). The aorta diameter measurement was taken at the narrowest section of the aortic root due to the use of continuous wave Doppler. Stroke volume was estimated as the product of VTI and aortic area, and Q was derived from the product of stroke volume and average heart rate measured during that sampling period. Systolic blood pressure (SBP) and diastolic blood pressure (DBP) were assessed on the left arm using a standard blood pressure cuff and stethoscope during the final minute of each workload. Mean arterial pressure was calculated as: DBP + 0.333 (SBP – DBP) and total vascular conductance was calculated as: Q (mL/min)/mean arterial pressure.

#### Femoral Artery Blood Flow and Limb Vascular Conductance

Femoral artery blood flow was measured in the right femoral artery at rest and during the last minute of each workload prior to the cardiac Doppler measurement. As per previous recommendations [24], the Doppler probe was placed below the inguinal ligament on the common femoral artery, 2–3 cm above the bifurcation of the superficial and profundus branches. To limit motion artefact during intense exercise, an immovable foam bar rested on the legs just above the knee and participants were able to hold stabilization bars on either side of the seat. Blood velocity (V) was calculated from the most distinct VTI indicating unimpeded flow within the artery [24]. Femoral artery area (A) was calculated from femoral artery diameter at each workload where A = (diameter/2)^2 ^× π. Blood flow at each load was calculated as limb blood flow = (V × A) × heart rate based on the recommendation of a previous investigation [25]. Limb vascular conductance was calculated as: limb blood flow (mL/min)/mean arterial pressure.

All measurements (heart and leg) were completed by the same trained sonography technician with 20 years of clinical experience and 10 years experience in heart and vascular exercise image measurement.

#### Muscle Oxygenation

Muscle oxygenation was measured with a NIRO 300 (Hamamatsu Photonics, Japan) spatially resolved near infrared oxygenation using the tissue oxygenation index (TOI). The TOI provides an average saturation of the haemoglobin volume present within the microvasculature and was determined with proprietary software available on the NIRO 300 based on the theoretical basis for differential pathlength in biological tissue and geometric distance between the light source and optode detector [26-28].

The probe was affixed in a black probe holder to ensure maintenance of distance between light source and detection probe. The probe was placed on the right leg within the distal position of vastus lateralis approximately 20 cm above the knee using standard recommendations for measurement during human exercise protocols [29]. The area was shaved to minimize any influence that hair may have had on light transmission and per previous recommendations [30] adipose tissue thickness (ATT) was measured with Harpenden skinfold calipers, to ensure that ATT was less than 1.5 mm (trained: 5.9 ± 1.2 mm; active: 9.7 ± 4.0 mm). Data were collected simultaneously and saved on-line at a sampling rate of 1 s utilizing a data acquisition system (Powerlab 16/30, ADInstruments, Colorado Springs, CO) and a desktop computer.

### Analysis

The primary measures of interest were assessed using a 3-way mixed model ANOVA [Inspired oxygen content (21 and 15%) × 5 intensities (rest, 25, 50, 75, 100%) × group (trained and active)]. Significance was set *a priori *at *p *< 0.05. Tukey post hoc comparisons were performed when significant main or interaction effects were observed. Data are presented as mean ± SD.

## Results

**Figure 1:**
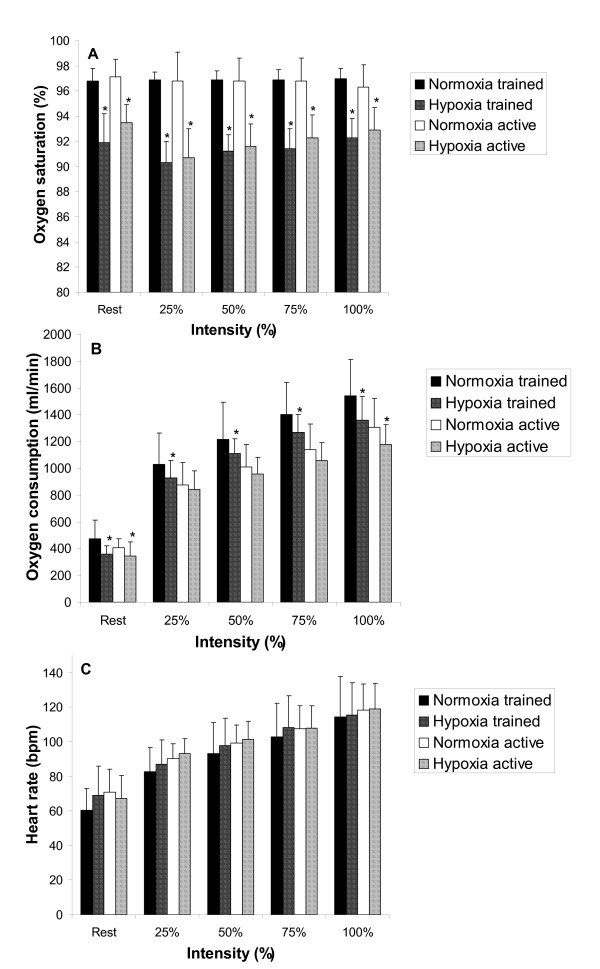
**Comparison of oxyhemoglobin saturation (Panel A), oxygen consumption (Panel B) and heart rate (Panel C) for trained and active groups at each intensity of exercise.** * means significantly different normoxia (*p *< 0.05).

There was a significant interaction amongst FIO_2 _condition and intensity and intensity and fitness for oxygen consumption. Specifically, hypoxia decreased VO_2 _at every workload in the trained group but VO_2 _only decreased at rest and 100% relative intensity in the active group (Figure 1B). A significant interaction between FIO_2 _condition and intensity for oxyhemoglobin saturation revealed significantly decreased oxyhemoglobin saturation in the hypoxic condition at every workload in both fitness groups (Figure 1A). The heart rate response was not different between FIO_2 _conditions (Figure 1C).

**Figure 2:**
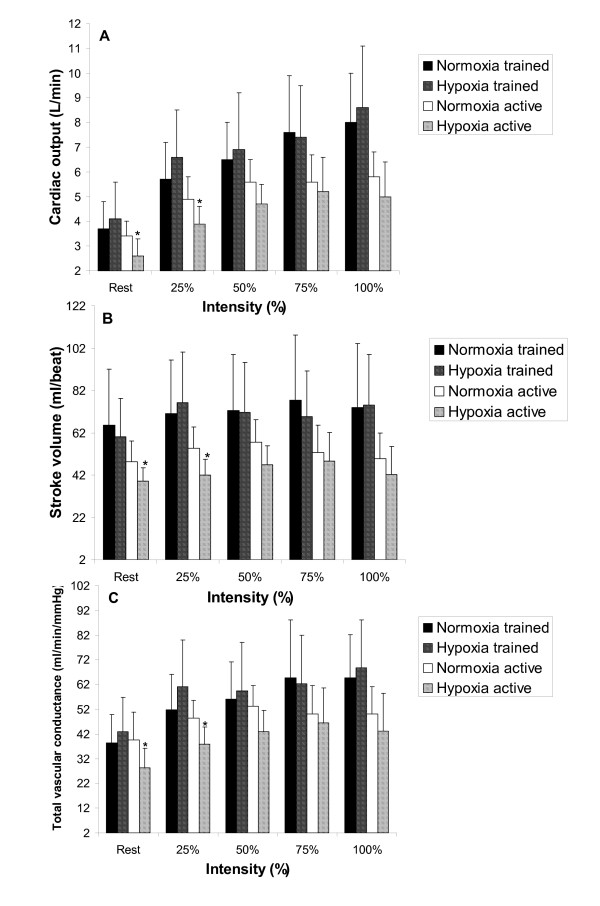
**Comparison of cardiac output (Panel A), stroke volume (Panel B) and total vascular conductance (Panel C) for trained and active groups at each intensity of exercise.** * means significantly different normoxia (*p *< 0.05).

The effect of hypoxia on the Q response at each workload for either group is shown in Figure 2A. There was a significant interaction amongst FIO_2 _condition, fitness and intensity for stroke volume and total vascular conductance which revealed decreases at rest and 25% relative intensity in the active group (Figure 2B and 2C respectively). The exercise response of ventilation and mean arterial pressure to hypoxia was no different than the normoxic response.

**Figure 3:**
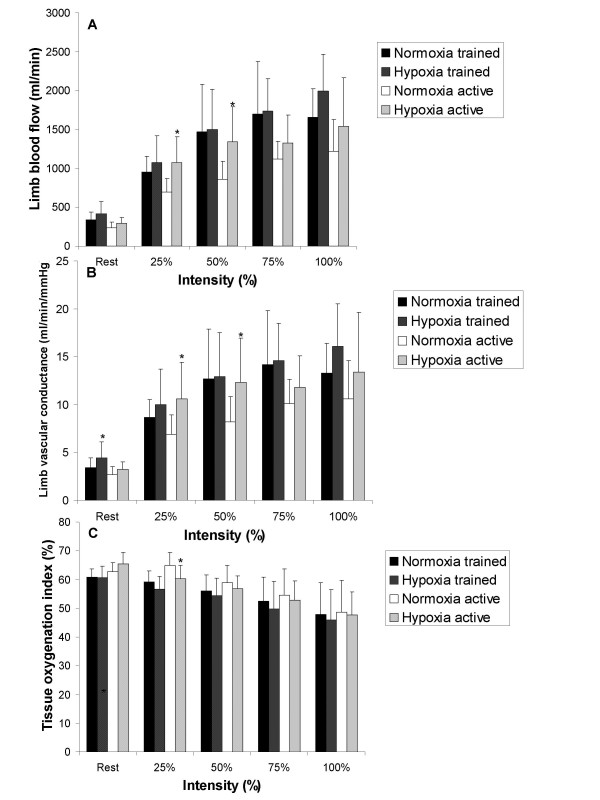
**Comparison of limb blood flow (Panel A), limb vascular conductance (Panel B) and tissue oxygenation (Panel C) for trained and active groups during exercise**. * means significantly different normoxia (*p *< 0.05).

There were no significant interactions for limb blood flow and limb vascular conductance however, post hoc analysis of the pairwise comparisons for limb blood flow and limb vascular conductance revealed that limb blood flow increased in hypoxia at 25 and 50% relative intensity in the active group and limb vascular conductance was greater in hypoxia at rest (trained group) and at 25 and 50% relative intensity (active group) (Figure 3A and 3B respectively).

Finally, there was a significant interaction between FIO_2 _condition and intensity for TOI, which revealed that hypoxia decreased TOI at 25% relative intensity in the active group (Figure 3C).

## Discussion

The major findings are that during sub-maximal small muscle mass exercise: 1) hypoxia elicits an increase in limb blood flow as well as a decrease in stroke volume and total vascular conductance in active males but does not significantly affect limb blood flow, conductance or Q in trained males; 2) at maximal exercise the trained group had similar Q and stroke volume in hypoxia and normoxia, but the active group decreased Q and stroke volume in hypoxia compared to the normoxic condition. In addition, both groups increased limb blood flow at maximum indicating a potential hypoxic stimulus during small muscle mass exercise. Overall this study provides a unique comparison between trained and active males to a hypoxic stimulus in localized muscle mass exercise and reflects different compensatory responses to hypoxic exercise that is dependent on the aerobic fitness of individuals.

### Rest and Sub-maximal Exercise

These results indicate that a hypoxic exercise stimulus elicits a limb blood flow increase compared to normoxia, at sub-maximal intensities (25%: + 378 mL/min, 50%: + 480 mL/min, 75%: + 210 mL/min; Figure 3A) in active males but not necessarily in trained males (Figure 3A). The enhanced blood flow for the active males was likely due to improved limb vascular conductance which was significantly greater at 25 and 50% relative intensity (+ 3.7 mL/min/mmHg and + 4.1 mL/min/mmHg; respectively) and 75% relative intensity (NS + 1.7 mL/min/mmHg Figure 3B). Previous investigations have found compensatory increases in limb blood flow at severe levels of hypoxia (FIO_2 _= ~11–12%) [1,31] in active males, however the current findings indicate that this form of compensation also occurs at a moderate level of hypoxia (FIO_2 _= 15%). Additionally, compensation in limb blood flow may not occur in well trained males during small muscle mass sub-maximal exercise (VO_2max _> 65 mL/kg/min) despite a similar decrease in oxygen saturation (Figure 1A) between the trained and active males.

There was no significant difference in muscle oxygenation between FIO_2 _conditions, unlike the results from other investigations [4,32] reporting an accelerated increase in deoxygenation to counter reduced arterial oxygen content. There was a small decrease in muscle oxygenation at each workload for both the active and trained groups (approximately 2 – 3%) compared to normoxia (Figure 3C). This would indicate that first, training status does not influence the deoxygenation response to a hypoxic exercise stimulus and secondly that compensation does not occur to a great extent within the muscle to maintain muscular work in a moderate hypoxic condition. However it remains to be clarified whether the muscle oxygenation response is proportional to the level of hypoxia (i.e. moderate hypoxia induces smaller change than severe hypoxia) not unlike blood oxygen saturation which decreases proportionately with decreasing FIO_2 _concentrations during cycling at the same intensity [33].

Comparison of systemic vascular conductance and heart function measures to the hypoxic condition reveals that active males decreased Q and total vascular conductance but trained males essentially maintained Q at rest and all sub-maximal intensities.

In the active males this equated to a 22% reduction in Q at rest in hypoxia compared to normoxic rest conditions paralleled by a decrease of 19% for SV. Considering that HR did not increase, the reduced SV predicated on a decreased TVC of 29% would explain why Q is reduced at rest in hypoxia in the active males. This is contrary to other who have found small increases in Q at rest in hypoxia compared to the normoxic condition [2,5] although acute short term hypoxic exposure (< 20 minutes) can result in increased diastolic blood pressure [34]. Secondary analysis of our results indicates that mean arterial pressure increased 8% at rest during hypoxia in the active males compared to the normoxic condition thus reduced Q (and SV) in our study at rest in the active males is likely attributed to a combination of increased mean arterial pressure and decreased TVC.

The trained males likely sustained similar Q due to a modest increase in stroke volume from rest to 25% (+ 16.1 mL/beat; Figure 2B), and a slightly elevated heart rate (Figure 1C). Interestingly VO_2 _during sub-maximal exercise was reduced throughout sub-maximal exercise in the trained group (p < 0.05) with a trend towards reduced VO_2 _in the active group (Figure 1B). This is an interesting finding especially in the trained group considering that the trained group's absolute workloads were approximately 35 – 40% > than the active group implying greater metabolic demand. Trained males have been shown to have a blunted hypoxic ventilatory response to exercise [35], and reduced oxyhemoglobin saturation and maximum heart rate [19] however, little else is known regarding the exercise response of trained and active males to hypoxia. It is understood that trained males possess reduced sub-maximal leg blood flow [36], and blood flow heterogeneity [37], which has a direct effect on improving muscular efficiency during exercise, however, these findings do not indicate why VO_2 _is significantly reduced in hypoxia for trained males. Systematic error in the measurement of VO_2 _during hypoxic exercise is a potential reason, although this investigation used a metabolic measurement system designed to be accurate at oxygen concentrations below normal physiological range. It is plausible that increases in anaerobic metabolism supplemented the reduced VO_2 _to maintain the same workload in hypoxia, yet the respiratory exchange ratio values were the same between FIO_2 _conditions. Nonetheless, further investigation of this finding is required including measurement of anaerobic energy contribution to elucidate whether this reduced VO_2 _is a valid hypoxic response.

### Maximum Exercise

At maximal exercise intensity in the hypoxic condition compared to the normoxic condition (active: 77.5 ± 11.0 watts, trained: 110.6 ± 21.3 watts), oxyhemoglobin saturation was reduced approximately 4.7% (trained) and 3.4% (active); with a reduced VO_2 _of approximately 200 mL/min (trained) and 125 mL/min (active). There was a trend towards decreased Q (0.8 L/min at maximum) in the active group attributed to a non-significant decrease in stroke volume (-7.3 mL). Reduced stroke volume was due in part to a reduced total vascular conductance (-6.8 mL/min/mmHg) which has been shown to affect stroke volume by increasing left ventricular afterload [38]. Conversely, the trained group had no notable changes in Q, SV or TVC however considering that hypoxia has been found to increase sympathetic vasoconstriction [39], these findings may reflect a training induced maintenance of oxygen delivery in hypoxia. Reasons for this maintenance may be due to significantly improved conductance during normoxic exercise for trained individuals compared to lesser trained individuals [40].

At the limb level, hypoxic exercise induced a non-significant increase in limb blood flow compared to normoxia in both groups (Figure 3A). Despite not being a significant increase, physiologically we believe this increase is meaningful and reflects a hypoxic stimulus to limb blood flow in small muscle exercise that has not been previously shown. This increase is likely predicated on enhanced limb vascular conductance (Figure 3B) that is approximately 21% (trained) or 25% (active) greater in hypoxia compared to normoxia. In contrast, others have shown a fall in limb blood flow during maximal KE exercise [1,2,41]. However, those investigations used a more severe level of hypoxia (FIO_2 _= 11–12%) than the level used in this study. Severe acute hypoxia (FIO_2 _= 11%) reduces work rate due to excessive fatigue such that the magnitude of the oxygen delivery is reduced [42]. In this study the participants were able to repeat the same exercise intensity in both conditions, likely as a result of the more moderate level of hypoxia imposed (FIO_2 _= 15%). Thus, the increased limb blood flow at maximum may be indicative of additional compensation to equalize oxygen delivery compared to normoxia. Within the muscle, this enhanced blood flow appears to moderate TOI so that oxygenation is not significantly changed when hypoxic conditions exist (decreased 1–2%; Figure 3C).

One other investigation has alluded to increased limb blood flow brought about by 1 leg KE maximal exercise in hypoxia (FIO_2 _= 10–11%) [3]. However, the absolute intensity was lower in hypoxia (approximately 5 watts less) and the determined normoxic maximum intensity was likely not a "true maximum". Despite these limitations, the results of Rowell et al. [3] and the present investigation are strongly suggestive of a hypoxic stimulus to increased limb blood flow during intense KE exercise. The physiological basis for this change is beyond the scope of this investigation however arterial oxygen content provides a reasonable hypothesis for future investigations. For instance, it has been recently shown that decreased saturation of haemoglobin has a direct influence on increasing both conductance and limb blood flow during exercise [31]. Moreover, regulation of skeletal muscle perfusion during exercise, likely involves adjusting blood flow relative to metabolic demand [43]. Because the workloads in this investigation were the same between FIO_2 _conditions, it can be assumed that metabolic demand within the muscle was consistent between normoxia and hypoxia, thus factors beyond metabolic control of muscle blood flow likely caused the increase in limb blood flow during the hypoxic condition. Thus the reduced blood saturation found in this study, suggests arterial oxygen saturation may be a mechanism by which vasodilatation occurs, thereby increasing muscle blood flow beyond the normoxic maximum value.

### Temporal Q and Limb Blood Flow Response

These results reflect an apparent underestimation of both limb blood flow and cardiac output due to a number of potential reasons described below. First, previous investigations have measured aorta diameter at the sinotubular junction and others at the aortic valve hinge points [44]. We used continuous wave Doppler, which does not allow for measurement of where "peak recorded velocity" occurred. Thus it is necessary to measure the aorta diameter at the point where theoretical peak velocity will occur, in this case the aortic valve hinge points. However, by recording the diameter measurement at the aortic valve hinge points, compared to the sinotubular junction a 43% reduction in diameter resulted.

In addition the KE protocol required significant muscular effort to extend the knee with little muscular effort on the return phase. This meant that at 75% relative intensity 37 and 53 kg and at 100% relative intensity 50 and 71 kg of weight was lifted at a rate of 40 contractions per minute in active and trained males respectively. This amount of weight required significant stabilization in the upper body which may have influenced the intra-thoracic pressure and atrial filling pressure reducing end diastolic volume and stroke volume. Thus, both of these factors likely influenced the magnitude of increase in Q that was lower than expected compared to other investigations using similar protocols and subject characteristics [45].

Furthermore, exercise limb blood flow values were lower than expected, despite resting leg blood flow data which was similar to others using both echocardiography and other techniques [3,4,46]. In comparison to others, the percentage of cardiac output that was targeted for the legs at maximum 2 leg knee extension exercise was approximately 42%, well below other published values [1,42,47]. This would indicate that something about the measurement during exercise had an influence on the leg blood flow calculation. The knee extension movement does produce deformation of the femoral artery which is evident during both low and high load work. Yet, it was clear when this was occurring on the echocardiography image, and no velocity time integrals were taken during these periods of time. Compared to the thermodilution technique which measures venous drainage from the entire limb [48], the echocardiography technique employed in this investigation measured only arterial blood flow targeted to a specific muscle group (quadriceps). In addition, femoral artery diameters were 2–3 mm smaller in both the active and trained groups compared to another investigation [24] yet neither of these differences completely resolves the large discrepancy still shown during exercise. Analysis of other exercise protocols [3,24,48] using similar participants reveals that cadence is generally greater than what was used in this investigation (60 contractions per minute compared to 40 contractions per minute in this investigation). In hindsight it is clear that success on the knee extension task required a significant strength component that was as important to maintaining cadence as oxygen delivery. As previous investigations has shown that increased cadence increases oxygen delivery during cycling at the same absolute work rate [49,50] the slower contraction cadence was likely the predominant factor in a reduced limb blood flow response compared to previous research.

### Application of Results to Performance

Altitude acclimatization has been a popular method of increasing blood haemoglobin concentration and arterial oxygen saturation, [51] however these enhancements on maximal exercise performance and VO_2max _remain limited [52]. Moreover, these adaptations to altitude in elite athletes have translated to small gains (1–2%) at sea level versus performance at altitude [12]. Recently, it has become apparent that gains in oxygen carrying capacity of blood due to chronic hypoxia are offset by reduced Q and redistribution of blood flow to non-exercising tissue [52], decreased total vascular conductance, increased circulating noradrenaline [53] as well as increased sympathetic tone after 13 days of heavy training (20–25 hrs/wk) while living at altitude [54]. These results would indicate that even during acute moderate hypoxia (equivalent to approximately 3000 meters) that some of these effects are more apparent in less well trained individuals, compared to highly trained individuals (VO_2max _> 65 mL/kg/min). With this understanding, it may be conjectured that endurance trained males may cope better during endurance performance events at moderate altitude however the comparative changes between trained and active individuals exposed to chronic hypoxia remain less clear.

## Conclusion

The effect of hypoxia on the cardiovascular response was evident during sub-maximal and maximal small muscle mass exercise while participants breathed 15% FIO_2_. These findings are an important distinction compared to other research (which in general has used FIO_2 _concentrations < 15%) because 15% FIO_2 _allowed all participants to complete the same workloads in both conditions. This allowed for a direct comparison of a hypoxic stimulus even during very intense exercise, where previous research has not yet adequately described this effect due to reduced maximal workloads compared to normoxia.

Application of these results to performance reveals that trained males may cope better at moderate altitude. Future research should confirm the existence of a hypoxic limb blood flow increase during maximal exercise, and the mechanism(s) which regulate this unique phenomenon.

## Competing interests

The authors declare that they have no competing interests.

## Authors' contributions

MDK designed the study, coordinated the study, acquired the data, performed the statistical analysis, and drafted the manuscript. DEW participated in coordination of the study, provided the equipment used in the study and provided important revisions to the manuscript. CAB participated in the design of the study, interpretation of the results, and preparation of the manuscript. BTE participated in acquisition of data and coordination of the study. JMS participated in acquisition of data and helped organize the study. MJH participated in the design of the study, interpretation of the results, and preparation of the manuscript. All authors have read and approved the final manuscript.

## References

[B1] Roach RC, Koskolou MD, Calbet JA, Saltin B (1999). Arterial O2 content and tension in regulation of cardiac output and leg blood flow during exercise in humans. Am J Physiol.

[B2] Koskolou MD, Calbet JA, Radegran G, Roach RC (1997). Hypoxia and the cardiovascular response to dynamic knee-extensor exercise. Am J Physiol.

[B3] Rowell LB, Saltin B, Kiens B, Christensen NJ (1986). Is peak quadriceps blood flow in humans even higher during exercise with hypoxemia?. Am J Physiol.

[B4] DeLorey DS, Shaw CN, Shoemaker JK, Kowalchuk JM, Paterson DH (2004). The effect of hypoxia on pulmonary O2 uptake, leg blood flow and muscle deoxygenation during single-leg knee-extension exercise. Exp Physiol.

[B5] Ainslie PN, Barach A, Murrell C, Hamlin M, Hellemans J, Ogoh S (2007). Alterations in cerebral autoregulation and cerebral blood flow velocity during acute hypoxia: rest and exercise. Am J Physiol Heart Circ Physiol.

[B6] Subudhi AW, Dimmen AC, Roach RC (2007). Effects of acute hypoxia on cerebral and muscle oxygenation during incremental exercise. J Appl Physiol.

[B7] Kawahara Y, Saito Y, Kashimura K, Muraoka I (2008). Relationship between Muscle Oxygenation Kinetics and the Rate of Decline in Peak Torque during Isokinetic Knee Extension in Acute Hypoxia and Normoxia. International Journal of Sports Medicine.

[B8] Clausen JP (1977). Effect of physical training on cardiovascular adjustments to exercise in man. Physiol Rev.

[B9] Roca J, Agusti AG, Alonso A, Poole DC, Viegas C, Barbera JA, Rodriguez-Roisin R, Ferrer A, Wagner PD (1992). Effects of training on muscle O2 transport at VO2max. J Appl Physiol.

[B10] Mollard P, Woorons X, Letournel M, Lamberto C, Favret F, Pichon A, Beaudry M, Richalet JP (2007). Determinants of maximal oxygen uptake in moderate acute hypoxia in endurance athletes. Eur J Appl Physiol.

[B11] Martin D, O'Kroy J (1993). Effects of acute hypoxia on the VO2 max of trained and untrained subjects. J Sports Sci.

[B12] Hahn AG, Gore CJ, Martin DT, Ashenden MJ, Roberts AD, Logan PA (2001). An evaluation of the concept of living at moderate altitude and training at sea level. Comp Biochem Physiol A Mol Integr Physiol.

[B13] Emonson DL, Aminuddin AH, Wight RL, Scroop GC, Gore CJ (1997). Training-induced increases in sea level VO2max and endurance are not enhanced by acute hypobaric exposure. Eur J Appl Physiol Occup Physiol.

[B14] Gore CJ, Hahn A, Rice A, Bourdon P, Lawrence S, Walsh C, Stanef T, Barnes P, Parisotto R, Martin D, Pyne D (1998). Altitude training at 2690m does not increase total haemoglobin mass or sea level VO2max in world champion track cyclists. J Sci Med Sport.

[B15] Gore CJ, Hahn AG, Aughey RJ, Martin DT, Ashenden MJ, Clark SA, Garnham AP, Roberts AD, Slater GJ, McKenna MJ (2001). Live high:train low increases muscle buffer capacity and submaximal cycling efficiency. Acta Physiol Scand.

[B16] Ferreira LF, Koga S, Barstow TJ (2007). Dynamics of noninvasively estimated microvascular O2 extraction during ramp exercise. J Appl Physiol.

[B17] DeLorey DS, Kowalchuk JM, Heenan AP, duManoir GR, Paterson DH (2007). Prior exercise speeds pulmonary O2 uptake kinetics by increases in both local muscle O2 availability and O2 utilization. J Appl Physiol.

[B18] Hoppeler H, Weibel ER (2000). Structural and functional limits for oxygen supply to muscle. Acta Physiol Scand.

[B19] Mollard P, Woorons X, Letournel M, Cornolo J, Lamberto C, Beaudry M, Richalet JP (2007). Role of maximal heart rate and arterial o2 saturation on the decrement of v.o2max in moderate acute hypoxia in trained and untrained men. Int J Sports Med.

[B20] Woorons X, Mollard P, Lamberto C, Letournel M, Richalet JP (2005). Effect of acute hypoxia on maximal exercise in trained and sedentary women. Med Sci Sports Exerc.

[B21] Saltin B, Blomqvist G, Mitchell JH, Johnson RL, Wildenthal K, Chapman CB (1968). Response to exercise after bed rest and after training. Circulation.

[B22] Macfarlane DJ (2001). Automated metabolic gas analysis systems: a review. Sports Med.

[B23] Warburton DE, Gledhill N, Jamnik VK, Krip B, Card N (1999). Induced hypervolemia, cardiac function, VO2max, and performance of elite cyclists. Med Sci Sports Exerc.

[B24] Radegran G (1997). Ultrasound Doppler estimates of femoral artery blood flow during dynamic knee extensor exercise in humans. J Appl Physiol.

[B25] Leyk D, Baum K, Wamser P, Wackerhage H, Essfeld D (1999). Cardiac output, leg blood flow and oxygen uptake during foot plantar flexions. Int J Sports Med.

[B26] HAMAMATSU PHOTONICS K.K. SD (1999). NIRO News No. 1.

[B27] Al-Rawi PG, Smielewski P, Kirkpatrick PJ (2001). Evaluation of a Near-Infrared Spectrometer (NIRO 300) for the Detection of Intracranial Oxygenation Changes in the Adult Head. Stroke.

[B28] Yoshitani K, Kawaguchi M, Tatsumi K, Kitaguchi K, Furuya H (2002). A Comparison of the INVOS 4100 and the NIRO 300 Near-Infrared Spectrophotometers. Anesth Analg.

[B29] McCully KK, Hamaoka T (2000). Near-infrared spectroscopy: what can it tell us about oxygen saturation in skeletal muscle?. Exerc Sport Sci Rev.

[B30] Quaresima V, Lepanto R, Ferrari M (2003). The use of near infrared spectroscopy in sports medicine. J Sports Med Phys Fitness.

[B31] Gonzalez-Alonso J, Richardson RS, Saltin B (2001). Exercising skeletal muscle blood flow in humans responds to reduction in arterial oxyhaemoglobin, but not to altered free oxygen. J Physiol.

[B32] Costes F, Barthelemy JC, Feasson L, Busso T, Geyssant A, Denis C (1996). Comparison of muscle near-infrared spectroscopy and femoral blood gases during steady-state exercise in humans. J Appl Physiol.

[B33] Woorons X, Mollard P, Pichon A, Lamberto C, Duvallet A, Richalet JP (2006). Moderate exercise in hypoxia induces a greater arterial desaturation in trained than untrained men. Scand J Med Sci Sports.

[B34] Gilmartin G, Tamisier R, Anand A, Cunnington D, Weiss JW (2006). Evidence of impaired hypoxic vasodilation after intermediate-duration hypoxic exposure in humans. Am J Physiol Heart Circ Physiol.

[B35] Guenette JA, Diep TT, Koehle MS, Foster GE, Richards JC, Sheel AW (2004). Acute hypoxic ventilatory response and exercise-induced arterial hypoxemia in men and women. Respir Physiol Neurobiol.

[B36] Proctor DN, Miller JD, Dietz NM, Minson CT, Joyner MJ (2001). Reduced submaximal leg blood flow after high-intensity aerobic training. J Appl Physiol.

[B37] Kalliokoski KK, Oikonen V, Takala TO, Sipila H, Knuuti J, Nuutila P (2001). Enhanced oxygen extraction and reduced flow heterogeneity in exercising muscle in endurance-trained men. Am J Physiol Endocrinol Metab.

[B38] Higginbotham MB, Morris KG, Williams RS, McHale PA, Coleman RE, Cobb FR (1986). Regulation of stroke volume during submaximal and maximal upright exercise in normal man. Circ Res.

[B39] Marshall JM (2000). Adenosine and muscle vasodilatation in acute systemic hypoxia. Acta Physiol Scand.

[B40] Klausen K, Secher NH, Clausen JP, Hartling O, Trap-Jensen J (1982). Central and regional circulatory adaptations to one-leg training. J Appl Physiol.

[B41] Richardson RS, Leigh JS, Wagner PD, Noyszewski EA (1999). Cellular PO2 as a determinant of maximal mitochondrial O(2) consumption in trained human skeletal muscle. J Appl Physiol.

[B42] Calbet JA, Boushel R, Radegran G, Sondergaard H, Wagner PD, Saltin B (2003). Determinants of maximal oxygen uptake in severe acute hypoxia. Am J Physiol Regul Integr Comp Physiol.

[B43] Delp MD, Laughlin MH (1998). Regulation of skeletal muscle perfusion during exercise. Acta Physiol Scand.

[B44] Rowland T, Obert P (2002). Doppler echocardiography for the estimation of cardiac output with exercise. Sports Med.

[B45] Di B, Santoro G, Talarico L, Di Muro C, Caputo MT, Giorgi D, Bertini A, Bianchi M, Giusti C (1996). Left ventricular function during exercise in athletes and in sedentary men. Med Sci Sports Exerc.

[B46] Radegran G (1999). Limb and skeletal muscle blood flow measurements at rest and during exercise in human subjects. Proc Nutr Soc.

[B47] Calbet JA, Jensen-Urstad M, van Hall G, Holmberg HC, Rosdahl H, Saltin B (2004). Maximal muscular vascular conductances during whole body upright exercise in humans. J Physiol.

[B48] Andersen P, Saltin B (1985). Maximal perfusion of skeletal muscle in man. J Physiol.

[B49] Ferreira LF, Lutjemeier BJ, Townsend DK, Barstow TJ (2006). Effects of pedal frequency on estimated muscle microvascular O2 extraction. Eur J Appl Physiol.

[B50] Gotshall RW, Bauer TA, Fahrner SL (1996). Cycling cadence alters exercise hemodynamics. Int J Sports Med.

[B51] Heinicke K, Heinicke I, Schmidt W, Wolfarth B (2005). A three-week traditional altitude training increases hemoglobin mass and red cell volume in elite biathlon athletes. Int J Sports Med.

[B52] Calbet JA, Boushel R, Radegran G, Sondergaard H, Wagner PD, Saltin B (2003). Why is VO2 max after altitude acclimatization still reduced despite normalization of arterial O2 content?. Am J Physiol Regul Integr Comp Physiol.

[B53] Calbet JA (2003). Chronic hypoxia increases blood pressure and noradrenaline spillover in healthy humans. J Physiol.

[B54] Cornolo J, Fouillot JP, Schmitt L, Povea C, Robach P, Richalet JP (2006). Interactions between exposure to hypoxia and the training-induced autonomic adaptations in a "live high-train low" session. Eur J Appl Physiol.

